# Alkaline Phosphatase as a Potential Biomarker of Muscle Function: A Pilot Study in Patients with Hypophosphatasia

**DOI:** 10.3390/ijms26136153

**Published:** 2025-06-26

**Authors:** María Carmen Andreo-López, Victoria Contreras-Bolívar, Luis Martínez-Heredia, Francisco Andújar-Vera, Diego Becerra-García, Trinidad González-Cejudo, Sheila González-Salvatierra, Cristina García-Fontana, Beatriz García-Fontana, Manuel Muñoz-Torres

**Affiliations:** 1 Endocrinology and Nutrition Unit, University Hospital Clínico San Cecilio, 18016 Granada, Spain; mcandreo21@gmail.com (M.C.A.-L.); victoriacontreras_87@hotmail.com (V.C.-B.); mmt@mamuto.es (M.M.-T.); 2 Instituto de Investigación Biosanitaria de Granada (Ibs.Granada), 18012 Granada, Spain; luismh95@gmail.com (L.M.-H.); diego.becerra.sspa@juntadeandalucia.es (D.B.-G.); trinigcej@hotmail.com (T.G.-C.); 3 CIBER on Frailty and Healthy Aging (CIBERFES), Instituto de Salud Carlos III, 28029 Madrid, Spain; sheila_gonzalezsalvatierra@hotmail.com; 4 Bioinformatic Unit, Instituto de Investigación Biosanitaria de Granada (Ibs. Granada), 18012 Granada, Spain; franciscoluisandujar@gmail.com; 5 Department of Computer Science and Artificial Intelligence, University of Granada, 18071 Granada, Spain; 6 Andalusian Research Institute in Data Science and Computational Intelligence (DaSCI Institute), 18007 Granada, Spain; 7 Nuclear Medicine Unit, University Hospital Clínico San Cecilio, 18016 Granada, Spain; 8 Clinical Analysis Unit, University Hospital Clínico San Cecilio, 18016 Granada, Spain; 9 Instituto de Investigación Biomédica de Málaga y Plataforma en Nanomedicina (IBIMA Plataforma BIONAND), 29590 Málaga, Spain; 10 Endocrinology and Nutrition Unit, Hospital Regional Universitario de Málaga, 29010 Málaga, Spain; 11 Department of Medicine, University of Granada, 18016 Granada, Spain

**Keywords:** alkaline phosphatase, hypophosphatasia, muscle strength and muscle mass

## Abstract

Alkaline phosphatase (ALP) deficiency has been linked to reduced physical performance, as seen in hypophosphatasia (HPP). However, its potential role in muscle function has not been fully explored. This was a cross-sectional study in 34 HPP adults and 34 matched healthy controls. Muscle strength was assessed using handgrip strength (HGS), considering values below the 10th percentile of the Spanish population as low strength. Muscle mass was evaluated using dual-energy X-ray absorptiometry and morphometric ultrasound. Bone mineral density (BMD) was measured at the lumbar spine, femoral neck, and total hip. The prevalence of low muscle strength was significantly higher in the HPP group compared to controls (30% vs. 6%; *p* = 0.009), with decreased HGS in the HPP group (*p* = 0.039). Positive associations were observed between ALP and femoral neck BMD, leg circumference, and fat-free mass and an inverse association with tricipital skinfold. Subjects with serum ALP activity below the sex-adjusted median had a significantly higher risk of low muscle strength independently of HPP diagnosis. ALP remained independently associated with HGS (*p* = 0.005), and a predictive model using ALP values showed strong capability to predict low-muscle-strength risk. Based on these results, we conclude circulating ALP levels are independently associated with muscle strength and may represent a useful biomarker for the early detection of muscle dysfunction. Future longitudinal or interventional studies are needed to assess whether ALP plays a causal role in muscle strength.

## 1. Introduction

Alkaline phosphatase (ALP) is a plasma membrane-anchored phosphomonoesterase that catalyzes dephosphorylation reactions of various substrates and is essential for PPi catabolism [1]. In humans, four ALP isoenzymes, encoded by four different genes, have been described: three of the ALP isoenzymes are tissue-specific (“intestinal,” “placental,” and “germ cell”) and one is tissue-nonspecific (TNSALP), the latter being mainly expressed in bone, liver, and kidney and accounting for approximately 95% of total serum ALP activity [2,3]. Dephosphorylation of inorganic pyrophosphate (PPi) and pyridoxal-5-phosphate (PLP), its main physiological substrates [3,4,5], is necessary for proper bone and tooth mineralization and for the synthesis of neurotransmitters in the central nervous system [6]. In addition to these functions, TNSALP performs other lesser-known functions due to the wide variety of substrates it is able to dephosphorylate. In fact, adults with hypophosphatasia (HPP)—a rare genetic disease characterized by deficient TNSALP activity due to mutations in the encoding gene—have a variety of symptoms, including reduced physical function, musculoskeletal pain, hypomineralization fractures, and dental abnormalities [7]. Although there is some evidence pointing to TNSALP influencing muscle function [6], research on the likely interrelationship between ALP levels and muscle function is scarce to date. Classically, muscle and bone strength have been biomechanically linked. Nevertheless, there are other lesser-known factors that seem to play a role in the regulation of muscle function. In HPP patients, different studies reported significant improvements in muscle function after a few months of treatment with recombinant TNSALP enzyme, despite minimal or undetectable changes in bone quality parameters [8,9,10]. Based on this, TNSALP may play a potential role in muscle physiology, and its deficiency may therefore be related to muscle dysfunction, a common feature observed in a significant proportion of HPP patients. In this context, TNSALP deficiency has been reported to disrupt mitochondrial function and lead to an increase in adenosine triphosphate (ATP) production in bone and muscle progenitor cells [11,12]. These alterations may contribute to the muscle impairments associated with HPP.

There is some evidence linking ALP activity to muscle. Specifically, high ALP values have been associated with sarcopenia in populations with a chronic pro-inflammatory state [13,14,15]. ALP plays a crucial role in regulating inflammation in various tissues [16,17], dephosphorylating microbial molecules, and regulating the proportion of adenine nucleotides to increase cyclic AMP levels, thereby inhibiting inflammatory processes such as neutrophil migration, reactive oxygen species (ROS) generation, and the production of pro-inflammatory cytokines [16,18,19]. Consequently, elevated ALP levels appear to exert an anti-inflammatory effect [16], so the elevation of ALP associated with sarcopenia in populations with chronic pro-inflammatory conditions could represent a protective mechanism to control inflammation, potentially influencing muscle function.

Based on these premises, it seems likely that there is a link between ALP and muscle function. Accordingly, the main objective of this study was to determine whether the ALP value can be useful as a marker of muscle function. Thus, the determination of ALP activity could be useful for early detection of probable sarcopenia or low muscle strength in the general population.

## 2. Results

### 2.1. Characteristics of the Study Population According to HPP

Table 1 summarizes the characteristics of the study population. Both groups were comparable in age, sex, and anthropometric characteristics. As expected, HPP patients showed significantly decreased ALP levels (66.5 IU/L (37–157) vs 26 IU/L (10–47); *p* < 0.001). The HPP group showed lower muscle strength (34 ± 13 Kg vs 29 ± 11 Kg, *p* = 0.039). In fact, the prevalence of low muscular function was higher in the HPP group (6% vs 30%; *p* = 0.009). However, there was no difference in muscle mass or bone parameters between the groups (except for femoral neck (FN) bone mineral density (BMD) which was significantly lower in the HPP group).

### 2.2. Correlations Between ALP and Muscle Strength and Mass in General

**Figure 1:**
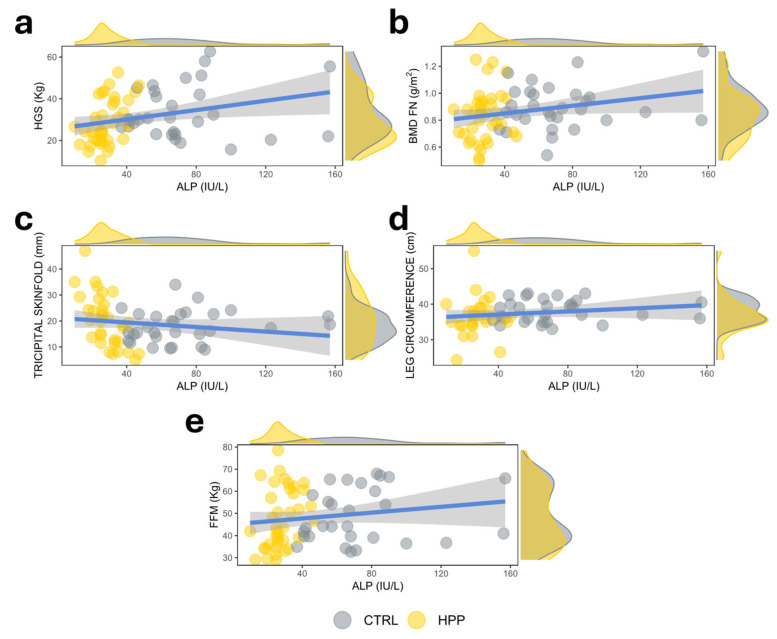
Scatterplot showing the correlation between ALP (IU/L) and HGS (Kg) in the study population. The *p*-values between the different associations were calculated using Spearman’s coefficient.The plots represent the correlations of ALP activity with (**a**) hand grip strength (HGS), (**b**) bone mineral density at the femoral neck (BMD FM), (**c**) tricipital skinfold, (**d**) leg circumference, (**e**) fat-free mass (FFM).

In the analysis of bivariate correlations, circulating ALP showed a positive association with handgrip strength (HGS) (r = 0.354; *p* = 0.003), BMD FN (r = 0.250; *p* = 0.041), leg circumference (r = 0.297; *p* = 0.025) and fat-free mass (FFM) (r = 0.247; *p* = 0.046) and a negative association with tricipital skinfold (r = −0.251; *p* = 0.047) (Figure 1a–e). However, serum levels of ALP did not correlate with ultrasound-determined muscle parameters: muscular area rectus anterior (MARA) (r = 0.013; *p* = 0.913), muscular circumference rectus (CMR) (r = −0.094; *p* = 0.450), X-axis (r = −0.060; *p* = 0.628), or Y-axis (r = 0.208; *p* = 0.089).

### 2.3. ALP Values According to Muscle Strength in the Study Population

**Figure 2:**
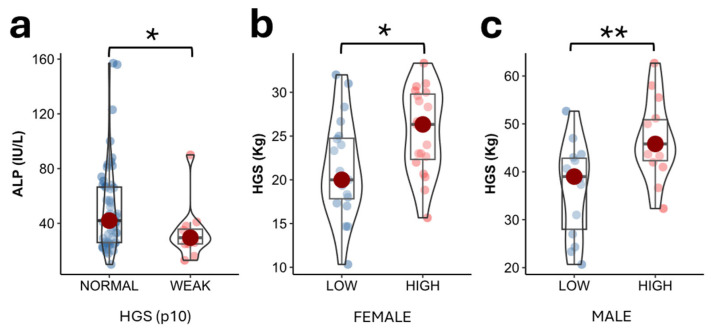
Serum ALP levels in the study population according to low or preserved muscle strength according to p10 of the Spanish population (**a**). HGS values in the study population according to levels below or above the median ALP adjusting for sex (**b**,**c**). The differences between groups were calculated using the Mann–Whitney test. Data are displayed as medians  ±  IQR. * *p*-value ≤ 0.05; ** *p*-value ≤ 0.01.

Circulating ALP levels were significantly higher in the group with preserved HGS, considering p10 of the Spanish population [20] as the reference cutoff point, compared to the group with decreased HGS (42.00 (40.5) IU/L vs 29.5 (10.8) IU/L; *p* = 0.047 (Figure 2a). When segregating the entire population by sex, circulating ALP levels below the median (31 IU/L for women and 43 IU/L for men) were associated with low muscle strength in females (20.0 (6.9) Kg vs 26.3 (7.5) Kg; *p* = 0.022) and males (39.0 (14.8) Kg vs 45.8 (8.6) Kg; *p* = 0.008) (Figure 2b,c).

### 2.4. Parameters Determining Muscle Strength in Study Population

To analyze the variables influencing HGS (dependent variable), a multiple backward linear regression model was performed, adjusting for variables associated in the bivariate analysis (ALP, fat-free mass index [FFMI], and percentage of fat mass [FM]), as well as other factors biologically linked to muscle strength, such as age. The results showed that FFMI (β  =  0.367; 95% CI [0.640–2.097]; *p*  <  0.001), %FM (β = –0.42; 95% CI [–0.858 to –0.312]; *p*  <  0.001), and ALP (β  =  0.24; 95% CI [0.025–0.163]; *p*  =  0.008) were independently associated with muscle strength (Table 2). The model showed a good fit, with an adjusted R² of 0.5037. Based on the VIF values, no multicollinearity was detected among the predictors (VIF: ALP = 1.00; FFMI = 1.25; FM = 1.25).

### 2.5. Effect of ALP on Muscle Strength in the Study Population

To identify low ALP levels as independent predictors of low muscle strength, a logistic regression using the maximum likelihood model was performed. For this purpose, low strength (values below the p10 of the reference values of muscle in the Spanish population adjusted for age and sex) was used as the dependent variable. The independent variables included in the model were sex-adjusted ALP cutoff point (model 1) and ALP cutoff point alongside FFMI due to its biological link with muscle function (model 2) (Table 3).

**Figure 3:**
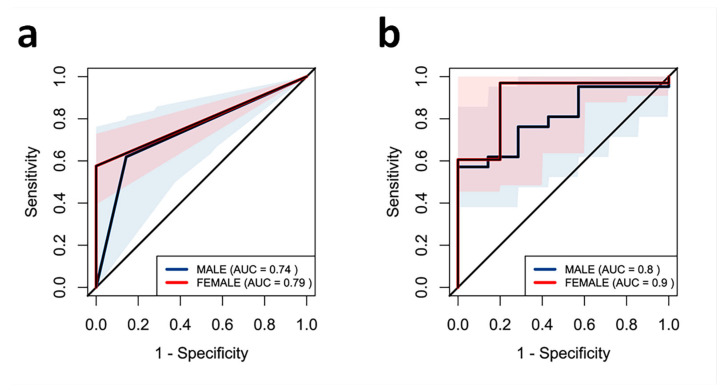
ROC curve for the usefulness of circulating levels of ALP in estimating risk of low muscle function in the study population:  0.79 for females (in red) and AUC  =  0.74 for males (in blue) (**a**). Full model including ALP levels and FFMI: AUC  =  0.8 for males (in blue) and AUC  =  0.9 for females (in red) (**b**). The AUC indicates the probability to predict an event, and values greater than 0.70 indicate good predictive performance. ROC: receiver operating characteristic; AUC: area under the curve.

ROC curve analysis was performed to assess the usefulness of low circulating ALP as a risk factor for low muscle strength. Two models were tested: one including only sex-adjusted ALP cutoff point (a) and other including the previous one, FFMI (b). The AUC for model A was 0.74 in males and 0.79 in females (Figure 3a). The area under the curve (AUC) for model B was 0.8 for males and 0.9 for females (Figure 3b).

## 3. Discussion

Our study explored the potential role of ALP as a biomarker associated with muscle strength. We observed a higher prevalence of low muscle strength in adults with lower ALP values and a positive association between ALP and muscle strength in both HPP and control subjects. Moreover, lower circulating ALP levels (below the median) were associated with an increased likelihood of low muscle strength in both men and women. To date, no biochemical biomarkers of muscle function have been firmly established. These findings suggest that ALP may be a promising candidate for future research on diagnostic and therapeutic strategies aimed at identifying individuals at risk.

Our results showed lower HGS values in HPP patients than controls. Accordingly, muscle and motor coordination deficiencies have been observed in *ALPL*−/− mice, as well as defects in physical functioning in HPP patients [7,11,21]. The mechanisms underlying the decline in muscle function are complex and not yet fully elucidated. Our findings suggest that serum ALP may play a more active role in muscle physiology than previously recognized. The consistent association between lower ALP levels and reduced HGS (observed even after stratifying by sex) raises the possibility that ALP is not merely a biochemical correlate, but may be biologically involved in maintaining muscle function. Reduced ALP activity could impair muscle energy supply, leading to diminished strength. These observations are particularly relevant in the context of HPP, where loss-of-function mutations in TNSALP result in chronically low ALP levels. This finding suggests that ALP could be the first biochemical marker of muscle function. Supporting this finding, our results also showed a positive correlation between ALP and validated muscle mass parameters, such as leg circumference and fat-free mass, in our study population. Additionally, an inverse correlation was observed between ALP and fat-related parameters that negatively impact muscle strength, such as tricipital skinfold. To date, no evidence is available relating ALP levels to muscle mass measured by calf circumference or fat mass measured by tricipital fold in a healthy population. Thus, our study is the first to explore and describe this interrelation. Conversely, no association was found between ALP levels and any muscle parameters assessed by ultrasound. Similarly, Hepp et al. found no significant differences in muscle mass parameters measured by DXA between an HPP group and healthy controls. However, no direct correlation between ALP and muscle or fat parameters was investigated [7]. In this study, muscle parameters were measured not only by reference techniques but also by muscle ultrasound to assess whether the results were comparable and extrapolable. Although ultrasound has potential and has shown promising results in the assessment of muscle mass, it is not validated for measuring muscle mass due to variability in operator technique, moderate correlation with reference methods, and lack of standardization in measurement protocols [22,23,24]. As such, further studies are needed to optimize the use of this technique for the evaluation of muscle parameters in clinical practice.

Our results show that circulating ALP alongside biologically linked variables such as FFMI and percentage of fat mass appear to be the main influencing variables of HGS. Nevertheless, the molecular mechanisms causing reduced physical function related to ALP deficiency remain unclear. One of the most studied hypotheses in recent years is about mitochondrial dysfunction and ATP excess in musculoskeletal cells secondary to ALP deficiency [11,12]. In this context, excess reactive oxidative species and impaired mitochondrial protein quality may directly affect muscle function [12]. Structural alterations of the mitochondria have been confirmed in animal models with low levels of ALP [14,15]. In HPP patients, persistently low circulating ALP levels may contribute to impaired muscle function through disrupted energy metabolism. Supporting this idea, Sun et al. demonstrated that genetic ablation of TNSALP in adipocytes rapidly induced an obese phenotype, highlighting the enzyme’s critical role in systemic energy regulation. Mechanistically, inhibition of TNSALP interferes with creatine cycling, which in turn compromises mitochondrial efficiency and reduces cellular energy output [25].

By contrast, some studies have reported an association between low muscle mass and elevated ALP levels in some populations. Lee JH et al. suggested that high ALP levels could act as predictors of sarcopenia, since they found a direct relationship between ALP tertiles and low skeletal muscle mass index in 15,579 South Korean adults from the Korea National Health and Nutrition Survey [13]. However, it is important to note that the circulating ALP levels reported in that study population for both men and women were significantly higher than the normal reference ranges established by the International Federation of Clinical Chemistry and Laboratory Medicine (33–98 IU/L in women and 43–115 IU/L in men). In that study, the first and third tertiles ranged between 200 and 250 IU/L in men and 170–224 IU/L in women. This suggests that the study population may have had secondary causes of hyperphosphatasemia and was clearly different from our study cohort. Likewise, other studies have reported a positive association between ALP levels and sarcopenia or poor physical performance in populations with liver cirrhosis and chronic hemodialysis. However, these conditions are characterized by a state of low-grade inflammation, which suggests that the elevated ALP levels observed in these populations may reflect a compensatory mechanism to counteract inflammation, given the known anti-inflammatory role of ALP rather than reflect muscle function [14,15]. In line with this, it has been proposed that elevated ALP levels could serve as an indirect marker of inflammation in these patients, since ALP activity increases under pro-inflammatory conditions [26,27]. Additionally, pro-inflammatory states may impair muscle strength through classical inflammasome activation pathways [28]. Finally, a cross-sectional study using NHANES 2011–2014 data [29] reported an inverse association between serum ALP levels and handgrip strength in healthy adults aged 40–80 years, particularly in those aged 40–59. The relationship was non-linear, with inflection points at 54 IU/L and 97 IU/L in men, suggesting a greater decline in muscle strength at higher ALP levels. These considerations suggest that the relationship between ALP and muscle function may not be linear across the full physiological spectrum. However, several factors may account for the discrepancies between this study and ours: while Zhang et al. analyzed an older, predominantly North American cohort, our study focused on a younger European/Hispanic population, in which this association has been largely unexplored. Moreover, the NHANES analysis did not include individuals with ALP levels below 48 IU/L, whereas our study focused specifically on this lower range, where we observed an association between reduced ALP activity and decreased muscle strength. These differences in population characteristics and analytical ranges may explain the divergent findings. Considering all this scientific evidence, it could be possible that both low and high ALP levels are linked to muscle dysfunction through distinct biological mechanisms, supporting the hypothesis of a potential U-shaped association between ALP activity and muscle strength, highlighting the need for further research across diverse cohorts and ALP activity ranges.

Our results also indicate that low circulating ALP levels (below the median) may independently predict the risk of low muscle strength. Specifically, ALP levels below 31 IU/L in women and 43 IU/L in men are associated with an approximately 13-fold and 7-fold increased risk of low muscle strength (below p10 of the reference values for the Spanish population), respectively. Furthermore, when FFMI is included in the model—given its statistical and biological association with HGS—the predictive accuracy improves even further (15- and 9-fold higher risk of low muscle strength in men and women, respectively). These findings support the potential utility of ALP, particularly in conjunction with body composition metrics, as part of a multi-marker approach to identify individuals at risk of functional decline. However, these ALP cutoffs should be interpreted cautiously, as they lack external validation and may be influenced by population-specific factors or overfitting, so further studies in independent cohorts are necessary to confirm their robustness and clinical applicability. However, and consistent with our findings, an ongoing international phase III clinical trial is currently evaluating the effectiveness and safety of enzyme replacement therapy with TNSALP in adult HPP patients who present with muscle weakness regardless of bone involvement based on emerging clinical evidence showing an improvement in generalized pain and motor function in Japanese adult and pediatric populations [8,9,10]. This reflects a growing recognition of the broader clinical spectrum of HPP and reinforces the observed link between ALP activity and muscle function, highlighting the clinical relevance of our results in the context of developing targeted therapeutic strategies. Nonetheless, it is important to note that further studies are needed in larger cohorts and populations to validate these results.

Our study has several strengths and limitations. One of the main limitations is its cross-sectional design, which prevents the establishment of a cause–effect relationship. Additionally, although our sample may be considered relatively small, it is more than acceptable given the low prevalence of HPP; however, the findings should not be extrapolated to broader populations without further studies. Moreover, certain functional tests, such as the 6 min walk test or the chair stand-up test, which could have provided additional insights into muscle function, were not performed. Furthermore, potential confounding factors such as physical activity levels or inflammatory markers were not evaluated in the study population. These variables will be considered in future research to provide a more comprehensive understanding of the observed associations. Despite these limitations, our study has notable strengths. To our knowledge, this is the first study to investigate the influence of ALP on muscle strength in both HPP patients and healthy individuals, shedding light on the complex interplay between bone and muscle. Moreover, our study population was well characterized, and we used multiple imaging techniques to explore new methodologies for sarcopenia assessment. These factors enhance the robustness of our findings and contribute to a better understanding of the potential role of ALP in muscle function, paving the way for future research exploring its clinical applications.

In conclusion, our findings suggest an association between circulating ALP levels and muscle strength, highlighting the potential of ALP as a biochemical marker worthy of further investigation. Notably, lower ALP levels were observed in individuals with reduced muscle function, pointing to its possible role as a predictor of low muscle strength. These associations emphasize the need for additional research to explore the clinical relevance of ALP in muscle function assessment and its potential application in the context of sarcopenia and related conditions.

## 4. Materials and Methods

### 4.1. Study Design and Recruitment of Participants

This was a cross-sectional observational study of 34 adult patients diagnosed with HPP and 34 healthy subjects matched by sex, age (±5 years), and body mass index (BMI) (±3 kg/m^2^) who were sequentially recruited at the Endocrinology Unit of the University Hospital Clínico San Cecilio of Granada (Spain) between October 2021 and May 2024. Subjects with low ALP levels caused by secondary hypophosphatasemia were excluded. All adults with HPP were diagnosed based on biochemical and/or clinical manifestations, and the diagnosis was genetically verified following the algorithm developed by García-Fontana et al. [30]. The study participants did not present with thyroid disease, disturbances in glucose homeostasis including diabetes mellitus, or evidence of nutritional deficiencies.

A post hoc analysis was conducted using G*Power 3.1 software to estimate the statistical power achieved when testing the null hypothesis of equality of proportions between two independent groups using a two-coiled Fisher’s exact test. Assuming a significance level of 5% and observed group proportions of 70% and 30%, with 34 participants in each group, the statistical power achieved was 87.59%.

### 4.2. Assessment of Muscle Functionality: Muscle Strength

Muscle strength was evaluated using an adult dynamometer (Jamar handgrip dynamometry, Asimow Engineering Co., Los Angeles, CA, USA), and was measured in the dominant limb, repeated on three occasions. The highest value was used to represent handgrip strength (HGS). Additionally, data from the Spanish population were used as reference values, establishing the cutoff point at percentile 10 (p10) [20].

### 4.3. Body Composition Measures

#### 4.3.1. Anthropometric Measures

Weight (kg) was assessed using a scale (SECA, Birmingham, UK), and height (m) was measured using a stadiometer (SECA, Birmingham, UK). Based on these two values, BMI was calculated (kg/m^2^).

#### 4.3.2. Body Composition by Dual Energy X-Ray Absorptiometry

Body composition was determined by dual-energy X-ray absorptiometry (DXA), a densitometer Lunar Prodigy Advance (General Electric Medical Systems), and the software EnCore 12.3 (iDXA and Prodigy Advance). All the scans were performed according to the manufacturer’s standard scan and positioning protocols. Weight and total and fat-free mass (FFM) were recorded [31]. In addition, FFMI was calculated (FFMI: fat-free mass in kg/height in m^2^) and the prevalence of FFM malnutrition was determined according to the European Society for Clinical Nutrition and Metabolism (ESPEN) criteria: <17 kg/m^2^ (men) or <15 kg/m^2^ (women) [32].

BMD at the lumbar spine (L1–L4), femoral neck, and total hip were also evaluated. The data are expressed in g/cm^2^ [31]. The diagnosis of low BMD based on chronological age or osteopenia/osteoporosis was established according to World Health Organization (WHO) criteria and International Society for Clinical Densitometry (ISCD) guidelines [33,34].

#### 4.3.3. Muscle Ultrasonography of the Quadriceps Rectus Femoris

Muscle ultrasonography of the quadriceps rectus femoris (QRF) was performed on the dominant lower extremity while the patient was in a supine position using a 10–12 MHz probe of a Sonosite W S-Nerve^®^ (Bothell, WA, USA) ultrasound. The probe was positioned perpendicularly to the longitudinal and transverse axis of the dominant QRF. The evaluation was performed without compression at the level of the lower third from the superior pole of the patella and the anterior superior iliac spine, measuring the X- and Y-axis (transverse muscle thickness), muscular circumference rectus (CMR), and muscular cross-sectional area rectus anterior (MARA) [35]. The ultrasounds were executed by the same person previously trained in this technique. Three measurements were taken for each parameter and the mean was calculated.

### 4.4. Biochemical Analysis

ALP activity was measured bichromatically at 410/480 nm by absorbance spectrophotometry using an AU5800 analyzer (Beckman Coulter) at the Clinical Analysis Laboratory of University Hospital Clínico San Cecilio of Granada. The conversion of p-nitrophenyl phosphate to p-nitrophenol in the presence of magnesium, zinc, and 2-amino-2-methyl-1-propanol as phosphate acceptor at pH 10.4 was determined from serum samples according to the method recommended by the International Federation of Clinical Chemistry. The reference values for adults adjusted by age and sex were those proposed by the International Federation of Clinical Chemistry and Laboratory Medicine [36].

Serum PLP concentrations were determined by high-performance liquid chromatography (HPLC) (Agilent 1200 Series isocratic HPLC system, Santa Clara, CA, USA) using a fluorescence detector (Agilent G1321A 1200 Series), and the sample preparation kit was provided by Chromsystems Instruments & Chemicals GmbH. The reference PLP values ranged from 3.6 to 18 ng/mL.

### 4.5. Statistical Analysis

Quantitative variables are presented as medians with interquartile range (IQR). Categorical variables are presented as percentages. The Shapiro–Wilk test was used to test the normality of distribution of the continuous variables. Covariate-adjusted (age) group comparisons were conducted using univariate analysis of covariance (ANCOVA). One-factor analysis of variance (one-factor ANOVA) was used to compare several groups. Categorical variables were compared using the χ^2^ test. Correlations between continuous variables were assessed using Spearman’s correlation coefficients. Multiple backward linear regression analysis was performed to identify independent variables associated with low muscle strength (dependent variable), including the quantitative and qualitative variables linked in the bivariate analysis, and other variables biologically associated with low muscle strength. Multicollinearity among predictors was evaluated using the variance inflation factor (VIF), with all included variables showing acceptable VIF values (<5). Adjusted R^2^ was used to assess the explained variance of the model. The standardized regression coefficient (B) was calculated considering 95% confidence intervals (Cis, lower limit/upper limit). To identify ALP as an independent predictor of low muscle strength (values below p10), logistic regression using the maximum likelihood model was performed, including ALP sex-adjusted cutoff as a dependent variable alongside FFMI as risk factors of low muscle strength. The usefulness of circulating ALP as an estimator of low-muscle-strength risk was assessed using a receiver operating characteristic (ROC) curve. The area under the curve (AUC) indicates the probability of predicting an event. AUC values greater than 0.75 indicate good predictive performance.

Statistical significance was set at *p* < 0.05 (two-tailed). The data analysis was performed using SPSS 26.0 (SPSS Inc., Chicago, IL, USA) and GraphPad Prism 7.03 (GraphPad Software).

## Figures and Tables

**Table 1:** Comparison of clinical, anthropometric, and musculoskeletal characteristics between the control and HPP groups.

	Controls (n = 34)	HPP (n = 34)	*p*-Value
**Clinical variables**			
Sex (% females)	56	56	0.761
Age (years)	49 (18–76)	50 (18–79)	0.968
ALP (IU/L)	66.5 (37–157)	26 (10–47)	<0.001 *
Muscle strength			
HGS (Kg)	30 (15–62)	25 (10–52)	0.039 *
Low muscle strength or dynapenia (%)	6	30	0.009 *
**Body composition parameters**			
BMI (Kg/m^2^)	25.8 (16.9–36.8)	25.6 (17.6–37.9)	0.662
FFM (DXA) (Kg)	44.28 (30–66.9)	43.57 (29–78.6)	0.882
Leg circumference (cm)	39.0 (33.0–44.5)	36 (24.3–55)	0.096
Tricipital skinfold (mm)	17.5 (9.0–34.0)	20.0 (5.0–47.0)	0.344
FFMI, females (Kg/m^2^)	15.5 (11.8–19.6)	14.84 (11.2–21.7)	0.551
FFMI, males (Kg/m^2^)	19.96 (16.1–21.4)	20.23 (17.2–25)	0.262
Low FFM (%)	11 (33)	11 (30)	0.634
FM (DXA) (%)	34.5 (15.9–49.3)	33.7 (14.5–48.3)	0.611
MARA (cm^2^)	3.76 (1.8–17.1)	4.15 (1.27–10.4)	0.551
CMR (cm)	8.25(6.64–16.3)	9.14(1.59–13.6)	0.262
X-axis (cm)	3.41 (1.02–5.71)	3.69(1.37–5.14)	0.634
Y-axis (cm)	1.41(0.82–3.12)	1.34 (0.8–3.74)	0.89
**Bone parameters**			
BMD (g/cm^2^)			
TH	0.95 (0.58–1.33)	0.98 (0.61–1.32)	0.469
FN	0.89 (0.54–1.31)	0.85 (0.50–1.25)	0.034 *
LS	1.12 (0.73–1.47)	1.06 (0.66–1.42)	0.16

Abbreviations: ALP: alkaline phosphatase; BMI: body mass index; FFM: fat-free mass; FFMI: fat-free mass index; FM: fat mass; DXA: dual-energy X-ray absorptiometry; CMR: muscular circumference rectus; MARA: muscular area rectus anterior; HGS: handgrip strength; CMB: bone mineral content; aBMD: areal bone mineral density; TH: total hip; FN: femoral neck; LS: lumbar spine. Data for continuous variables are presented as medians followed by interquartile ranges in brackets. Data for categorical variables are presented as percentages. Comparisons between the groups were conducted by ANCOVA, adjusting for sex. * Significant differences between groups (*p* ≤ 0.05).

**Table 2:** Linear regression analysis indicating variables independently associated with HGS. Variables included in the original model were age, FFMI, FM, and ALP. Level of significance <0.05.

	Non-Standardized Coefficients				
	B	Error Typ.	Beta	95% CI	*p*-Value
HGS					
FFMI	1.368	0.364	0.367	0.640–2.097	<0.001
% FM	−0.585	0.137	−0.42	−0.858–(−0.312)	<0.001
ALP	0.094	0.034	0.24	0.025–0.163	0.008

Abbreviations: HGS: handgrip strength; FM: fat mass, FFMI: fat-free mass index, ALP: alkaline phosphatase; CI: confidence interval.

**Table 3:** Maximum likelihood model of logistic regression for low-muscle-strength risk.

**Model 1**			
***Variables***	***Exp (B)***	***CI 95%***	***p-Value***
ALP cutoff, male	6.88	1.15–75	0.034 *
ALP cutoff, female	13.13	1317–1775.01	0.018 *
**Model 2**			
***Variables***	***Exp (B)***	***CI 95%***	***p-Value***
ALP cutoff, male	9.25	1.31–120.47	0.024 *
FFMI	1.18	0.79–1.87	0.428
ALP cutoff, female	14.84	1.49–2007.6	0.018 *
FFMI	1.23	0.82–2.2	0.347

Dependent variables: sex-adjusted ALP cutoff point (<31 UI/L for females; <43 IU/L for males) (model 1) and sex-adjusted ALP cutoff point alongside FFMI (model 2). * Significant differences between groups (*p* ≤ 0.05).

## Data Availability

All the data used for the analyses in this report are available from the corresponding author upon reasonable request.

## Abbreviations

The following abbreviations are used in this manuscript:

ALP	alkaline phosphatase
TNSALP	tissue-nonspecific alkaline phosphatase
PPi	inorganic pyrophosphate
HPP	hypophosphatasia
ATP	adenosine triphosphate
ROS	reactive oxygen species
BMD	bone mineral density
BMI	body mass index
FFM	fat-free mass
FFMI	fat-free mass index
FM	fat mass
DXA	dual-energy X-ray absorptiometry
CMR	muscular circumference rectus
MARA	muscular area rectus anterior
HGS	handgrip strength
CMB	bone mineral content
aBMD	areal bone mineral density
TH	total hip
FN	femoral neck
LS	lumbar spine
